# Agricultural machinery automatic navigation technology

**DOI:** 10.1016/j.isci.2023.108714

**Published:** 2023-12-14

**Authors:** Zhixin Yao, Chunjiang Zhao, Taihong Zhang

**Affiliations:** 1 College of Computer and Information Engineering, Xinjiang Agricultural University, Urumqi 830052, China; 2 National Engineering Research Center for Information Technology in Agriculture, Beijing 100083, China; 3 Engineering Research Center of Intelligent Agriculture, Ministry of Education, Urumqi 830052, China

**Keywords:** Applied sciences, Agricultural science, Agricultural engineering, Engineering

## Abstract

In this paper, we review, compare, and analyze previous studies on agricultural machinery automatic navigation and path planning technologies. First, the paper introduces the fundamental components of agricultural machinery autonomous driving, including automatic navigation, path planning, control systems, and communication modules. Generally, the methods for automatic navigation technology can be divided into three categories: Global Navigation Satellite System (GNSS), Machine Vision, and Laser Radar. The structures, advantages, and disadvantages of different methods and the technical difficulties of current research are summarized and compared. At present, the more successful way is to use GNSS combined with machine vision to provide guarantee for agricultural machinery to avoid obstacles and generate the optimal path. Then the path planning methods are described, including four path planning algorithms based on graph search, sampling, optimization, and learning. This paper proposes 22 available algorithms according to different application scenarios and summarizes the challenges and difficulties that have not been completely solved in the current research. Finally, some suggestions on the difficulties arising in these studies are proposed for further research.

## Graphical abstract

**Figure:**
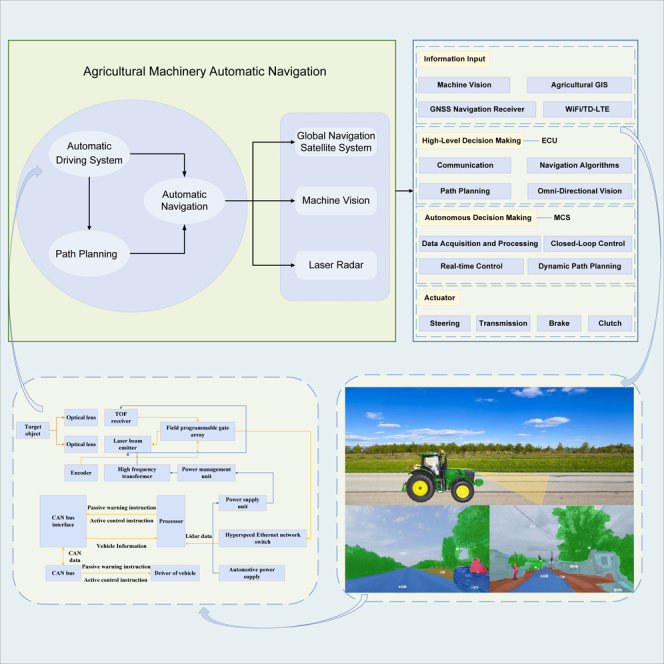

## Introduction

Smart agriculture (SA) is a crucial direction for the future of agricultural development. However, to realize true SA, it is essential to first achieve informatization and intelligence in agricultural machinery. In recent years, major agricultural nations worldwide have invested substantial resources and manpower into the research of intelligent agricultural equipment and machinery. As a result, an increasing number of technologies related to autonomous agricultural machinery have been developed, bringing together knowledge from various disciplines. These advancements not only enhance land productivity, labor efficiency, and resource utilization but also contribute to increased profitability for farmers. The integration of sensors with agricultural machine vision and image processing techniques has become a critical aspect of the intelligence, automation, and sophistication of agricultural machinery. Autonomous driving technology for agricultural machinery helps reduce labor intensity, lower input costs, and boost profitability, providing a foundational support for farmers to generate income and engage in large-scale operations.

At present, there is a serious shortage of rural labor, and labor costs are increasing day by day. In the past, agricultural production methods relying on manual labor can no longer meet the needs of agricultural development. Based on interviews with 40 precision farmers and 40 traditional banana farmers in India, Franco^1^^,^^2^ found that land size has the highest positive impact on SA adoption. Balogh^3^ conducted 604 interviews and 30 semi-structured interviews in Hungary. They also found that larger farm sizes are a means of faster adoption and spread of SA. Jaafar and Kharroubi^4^ interviewed 577 farmers in Lebanon and found that younger farmers have a higher acceptance of SA. Most of the farmers were willing to try free mobile smart irrigation systems. According to the survey, Lachia^5^ found that there are currently about 150 self-guided weeding robots in use on sugar beet and vegetable farms in France. Rial-Lovera^6^ conducted interviews with 14 experts in California, USA, and the results show that the shortage of agricultural labor is a key factor in the development of autonomous driving in agricultural machinery.

SA plays a huge role in five aspects: agricultural production, agricultural management, agricultural harvest market, sustainable agricultural development, and rural social governance.^7^ Through the digital agriculture, agricultural big data in the Internet of Things, cloud computing, and emerging technologies such as artificial intelligence optimize and upgrade the traditional manual mode of agricultural production. It can not only improve production efficiency and reduce labor costs but also improve the quality of agricultural products and strengthen the sustainable development of agriculture. In agricultural production, farmland is generally managed delicately through remote sensing, sensors, Geographic Information System (GIS),^8^ and other technologies. The use of artificial intelligence technology to control precision fertilization and water and soil irrigation can achieve the purpose of saving water and fertilizer costs. In agricultural machinery automation applications, usually using intelligent robots, intelligent sensing technology is used to realize the automatic navigation of agricultural machinery and field assignments independently. This method can improve the operation efficiency of agricultural machinery, reduce the operation cost, and save the operation time. In the information management of agricultural products, the planting information and sales price of agricultural products are input through Radio Frequency Identification (RFID),^9^ quick response code, and other technologies. Implementing precise QR code recognition on the internet not only facilitates centralized management of agricultural product information for farmers but also helps consumers quickly purchase safe and reliable agricultural products, thereby enhancing the market competitiveness of agricultural products. Thus, intelligent agriculture is the inevitable trend of the future agricultural development. With the innovation of science and technology, the development prospect of wisdom agriculture will be broader. In order to alleviate the shortage of rural labor resources, it is particularly important to realize the automatic driving of agricultural machinery, improve the intelligent level of agricultural machinery, and reduce the demand for agricultural labor.

At present, the related research on autonomous driving focuses on multiple disciplines, such as artificial intelligence, control theory, navigation, and positioning. In this paper, the main task is to study agricultural machinery automatic navigation of autonomous and path planning technology. Through automatic navigation and path planning methods, the efficiency of agricultural production can be effectively improved and the waste of energy and resources can be reduced, which has a positive impact on sustainable development.^10^ This not only contributes to the modernization of agricultural production but also lays the foundation for sustainable agricultural development. Based on the existing relevant literature on intelligent agricultural machinery research, this paper reviews the research status, challenges, and solutions of automatic navigation technology for agricultural machinery. First, starting from the three different ways of agricultural machinery automatic navigation. Taking Global Navigation Satellite System, machine vision, and laser radar as the main classification, the related research of various scholars is introduced, and their advantages and disadvantages are summarized. And then the research status of agricultural machinery automatic navigation path planning algorithm is discussed. The development trend of four main research methods of path planning is studied. It provides reference for the research of automatic driving agricultural machinery in intelligent agriculture.

## Research progress on automatic driving technology of agricultural machinery

Agricultural automatic driving technology has been widely used in the field of SA. This method can improve the service life of agricultural machinery and reduce the cost of agricultural machinery. And it can reduce the working time of farmers and reduce the harm caused by continuous operation fatigue driving. This method can achieve 24-h automatic driving regardless of weather, region, and time. In the future, automatic driving technology can also be applied to forestry, animal husbandry, grassland management, and other fields to further improve the development space of automatic driving technology.

### The composition of agricultural machinery automatic driving system

**Figure 1:**
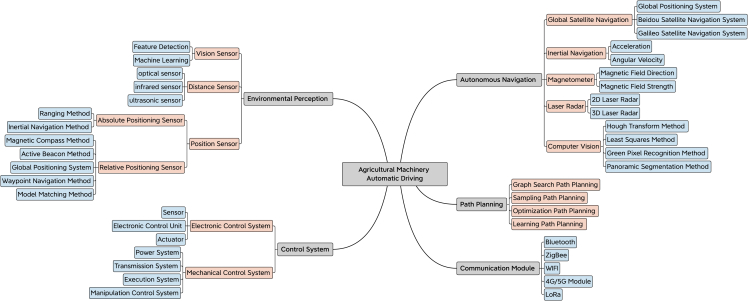
Agricultural machinery automatic driving system

Agricultural machinery automatic driving system is a very complex system, which includes agricultural machinery automatic navigation, path planning,^11^ control system, and communication module. The composition structure of the agricultural machinery automatic driving system is shown in Figure 1. This paper adopts a top-down approach to introduce the various components of agricultural machinery and briefly describes the technical difficulties of current research. Then it focuses on the automatic navigation module of agricultural machinery and the path planning algorithm to solve the problem of autonomous obstacle avoidance of agricultural machinery, which will be elaborated upon in the subsequent sections. Automatic navigation of agricultural machinery is a crucial step in the automatic driving system of agricultural machinery. It mainly relies on machine vision, laser radar, and GPS technology to identify and analyze the surrounding environment, so that it can normally drive on rural roads. Path planning is the key algorithm to realize the autonomous obstacle avoidance function in agricultural machinery automatic navigation. It can select the optimal route for agricultural machinery driving, not only reduce the time cost and energy consumption but also avoid the collision of agricultural machinery and improve navigation safety. This method includes global path planning and local path planning,^12^^,^^13^ and it usually uses graph-search-based path planning, sampling-based path planning, optimization-based path planning, and learning-based path planning methods for calculation. The control system refers to the electronic control system and mechanical control system of agricultural machinery, which is responsible for executing the instructions of path planning and navigation. The communication module includes the use of Bluetooth, ZigBee, WIFI, Narrowband Internet of Things (NB-IoT), Long Range (LoRa), and other technologies to realize the communication interaction of the Internet of Things. They are briefly introduced in *Control system* and *Communication module*. Firstly, the current geographical position, driving direction, and driving speed are determined by agricultural machinery automatic navigation technology. And then the path planning algorithm is used to achieve autonomous obstacle avoidance and calculate the optimal path in the current geographical environment. The basic information of the optimal path is sent to the agricultural machinery automatic driving system through the communication module, and the task instructions and real-time data of the system are received. Then the information is sent to the control system. When the control system receives the command, it begins to execute the navigation task. The steering and speed of the navigation are controlled by PID and other operations to ensure that the agricultural machinery can run normally according to the planned path.

#### Control system

The control system usually includes two parts: electronic control system and mechanical control system. The collaborative operation of these two systems enables agricultural machinery to achieve autonomous navigation and execute tasks independently. The electronic control system consists of sensors, electronic control units, and actuators. The mechanical control system comprises the power system, the transmission system, the execution system, and the handling control system. This system is capable of making decisions for path planning tasks based on real-time sensor data. It achieves functions such as obstacle avoidance, adjustment of machinery speed, and adaptation to various geographical conditions, ensuring the proper execution of instructions by the agricultural machinery. As shown in Table 1, Li^11^ designed and developed a master-slave automatic navigation control system for agricultural machinery. The master unit offers both automatic and manual driving modes, whereas the slave unit follows the master unit to carry out autonomous navigation tasks. The automatic navigation system adopts a steering wheel control method and consists of a positioning unit, steering control device, wireless communication unit, and vehicle terminal device. Experiments were conducted with a pilot battery car as the master unit and the Euro-leopard M904-D tractor as the slave unit, resulting in a root-mean-square error of 6.76 cm. However, this master-slave control system is not suitable for automatic navigation of large parcels, and the cost is high. In contrast, in 2020, Kanagasingham^14^ developed a control system based on an automatic weeding robot that combines GNSS, a compass, and computer vision. This system exhibited relatively lower errors and a more cost-effective solution. An average deviation of 4.59 cm was achieved without damaging the field crops. However, when the density of weeds in the field is high, the navigation accuracy of the control system will be reduced without harming the crops. Furthermore, the author only carried out experiments on one type of agricultural machinery. Although the navigation accuracy was relatively high, the universality of the control system remains to be verified, as it lacks testing on other types of agricultural machinery. Compared with the previous two methods, Yin^15^ developed an automatic navigation controller, which was tested on three different types of wheeled vehicles, namely, a rice transplanter, a sprayer, and a tractor. This controller incorporates functions such as automatic steering, hydrostatic transmission, and speed control. Through the GNSS combined with path planning algorithm in the field experiment, it was found that the maximum lateral root-mean-square errors for the rice transplanter, sprayer, and tractor’s autonomous navigation did not exceed 3.10 cm, 4.75 cm, and 2.21 cm, respectively. It shows that the control system has good robustness in different types of agricultural machinery automatic navigation.

**Table 1:** Application of control system in automatic driving of agricultural machinery

Reference	Research object	Evaluation metric	Error range/cm
Li^11^	Master-slave automatic navigation control system	RMSE	6.76
Kanagasingham^14^	Automatic weeding robot	MD	4.59
Yin^15^	Rice transplanter	RMSE	3.1
	Sprayer	RMSE	4.75
	Tractor	RMSE	2.21

#### Communication module

The communication module includes Bluetooth, ZigBee, WiFi, NB-IoT, and LoRa, among others. These devices enable the transmission of task progress and real-time data to the agricultural machinery’s autonomous driving system. This allows the control system to monitor the machinery’s operational status in a timely manner. In general, the protocols used for data transmission include TCP/IP, MQTT, HTTP, Modbus, and others. These specific communication protocols ensure the effective transmission of data and error detection. Feng^16^ proposed three viable wireless communication technologies, namely Wireless Sensor Networks (WSN) architectures based on NB-IoT, LoRa, and ZigBee. The experimental comparison proves that these three methods can communicate within acceptable time frames, but ZigBee is more suitable for use in facility agriculture, which can reduce the power consumption of wireless communication. NB-IoT and LoRa are more suitable for automatic navigation wireless communication technology in the field of agricultural scenarios. Ouyang^17^ introduced a solution that combines RTK-GNSS with an automatic delivery and recovery system based on WSN nodes. It helps agricultural machinery to deal with the problem that communication data are huge and difficult to maintain and adjust in real time during automatic navigation. The feasibility of this method was verified both indoors and outdoors. Furthermore, the feasibility of the traditional GPS+Compass combination was ruled out, as its accuracy did not meet commercial requirements and was unsuitable for wireless communication in complex agricultural machinery autonomous navigation scenarios. Tao^18^ also conducted a comprehensive search from three databases: ScienceDirect, IEEE Xplore, and Scopus. He summarized the current development of Internet of Things (IoT) communication technology in SA, analyzed the sensor detection equipment and communication technology involved in agricultural machinery autonomous navigation, and put forward some feasible schemes for researchers to reference, for the realization of agricultural machinery automatic navigation laid a certain foundation.

### The challenges

There are two challenges in rural road agricultural machine automatic driving technology, including technology and accuracy. From the technical level, the rural road scene is complex and changeable. In the process of automatic driving, agricultural machinery needs to carry out environmental perception and path planning for different terrains and different crop vegetation under different weather conditions. There is still a certain gap between the accuracy and reliability of the current environment awareness technology and the reality. This is prone to accidents. Therefore, sensors such as depth camera and millimeter wave radar can be used to obtain information and data to improve the safety of agricultural machinery automatic driving. Secondly, path planning is mostly through the combination of GPS positioning and vision sensors. Using traditional machine learning algorithms for planning has certain limitations. And the recognition effect is poor when the base station signal is unstable. The deep learning algorithm in artificial intelligence technology can be used to select the optimal path. This can make the automatic driving of agricultural machinery more intelligent.

From the perspective of accuracy, the biggest problem faced by autonomous driving is to achieve accurate positioning. To achieve high precision mapping and positioning, it is necessary to reduce sensor errors and improve the accuracy of the map. More advanced positioning and sensor technologies such as RTK-GPS, lidar, and vision sensors can be used.

## Research progress on automatic navigation technology of agricultural machinery

Agricultural machinery automatic navigation technology mainly refers to the agricultural machinery with the help of environmental perception system to feedback its position information to the automatic driving system^19^ in order to ensure that agricultural machinery can make the right decision according to the environment in the process of operation and driving.

### GNSS automatic navigation technology for agricultural machinery

**Figure 2:**
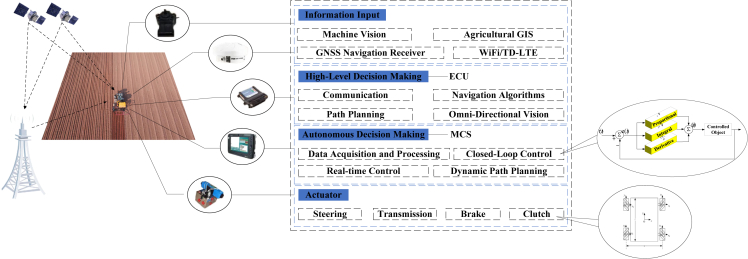
Agricultural machinery automatic navigation control system

GNSS technology uses multiple satellites around the Earth to accurately position a point to be measured, so as to obtain the absolute position of the point in geographical space. The positioning method includes single point positioning and differential positioning.^20^ The single point positioning uses the data of GNSS receiver to locate the point to be measured. In contrast, differential positioning utilizes more than two GNSS receivers simultaneously. Therefore, the latter has higher accuracy. The agricultural machinery automatic navigation control system is shown in Figure 2.

Automatic driving of agricultural machinery requires high perception accuracy and typically uses the differential positioning method. At present, scholars in various countries have made different attempts on agricultural machinery according to different fusion models. As shown in Table 2, O'Connor^21^ from Stanford University first applied the Carrier-phase Differential Global Positioning System (CD-GPS) based on four antennas to tractor navigation. It is tested on the John Deere 7800 tractor. It is verified that the heading Angle error is less than 0.1° and the lateral deviation is less than 2.5cm when the tractor runs in a straight line at a driving speed of 3.25 km/h. Benson^22^ designed a tractor navigation System based on Differential Global Positioning System (DGPS). The average lateral deviation of this navigation system is less than 1 cm when the tractor moves in a straight line. On the basis of the previous work, Bell^23^ designed the agricultural machinery navigation system based on the carrier phase difference global positioning system. It is proved that this type of GPS can be applied to commercial tractor navigation system. Nørremark^24^ has also developed a self-propelled mower system using GPS technology. And the Kalman filter is used to process the signal to further improve the positioning accuracy of the system. Gomez-Gil^25^ provides accurate positioning information for tractors by fusing GPS positioning data and tractor dynamic model. Experimental results show that the positioning accuracy of this method is significantly improved compared with the single GPS positioning method. Ortiz^26^ used real-time kinematic difference global positioning system (RTK-GPS) to realize the automatic navigation of the peanut harvesting machine. The results show the advantages of the used method over manual driving operations. Bakker^27^ developed an RTK-GPS-based automatic navigation method for agricultural machinery applied to sugar beet fields. It realizes autonomous navigation in rural roads, and the positioning accuracy reaches the centimeter level. Samuel^28^ designed a GPS smart farm machine that integrates multiple sensors. It can reduce the influence of weather, terrain, obstacles, and other variable factors on the positioning accuracy. Zhang^29^ took the rice transplanter in Kubota as the research object and used RTK-GPS and electronic compass for positioning. It realizes the control of the transplanter on the curve and straight path. And it is verified that the maximum lateral deviation is less than 8.7 cm when the transplanter runs at a speed of 0.33 m/s. Luo^30^ designed a navigation control system based on RTK-GPS and tested it on the Dongfanghong X-804 tractor. The experimental results show that the system has high accuracy, and the lateral deviation is less than 15 cm when the tractor’s forward speed is 0.8 m/s. Wei^31^ used the RTK-GPS positioning method to conduct experiments on a high-speed rice transplanter. When the forward speed is less than 2.16 km/h, the maximum lateral error is less than 10 cm. Ren^32^ and Guo^33^ used differential GPS technology to test different types of rice transplanter and realized the straight-line path navigation of rice transplanter in field operation. Xiong^34^ used the RTK-BDS positioning method based on the Beidou satellite navigation system to realize the automatic navigation and spraying operation of the orchard pesticide applicator. Liu^35^ applied the RTK-GNSS technology to the automatic navigation system of the ZP9500 spray machine and tested it in the cement field, dry field, and paddy field. The maximum lateral deviation of the system is 17.5cm in the extremely complex operation environment such as paddy field.

**Table 2:** Automatic navigation technology based on GNSS

Key methods	Research object	Error range/cm
CD-GPS^21^	Straight driving	2.5
DGPS^22^^,^^23^^,^^24^^,^^25^	Straight driving	1
RTK-GPS^26^^,^^27^^,^^28^	Business navigator	1
RTK-GPS+Compass^29^^,^^30^^,^^31^	Kubota rice transplanter	8.7
RTK-BDS^32^^,^^33^^,^^34^	Orchard applicator	10
RTK-GNSS^35^	Test of cement, dry land, and paddy field in complex environment	17.5

Technical difficulties:

(1) Due to the complex and changeable rural road environment, it is difficult to plan a safer navigation path. Therefore, these methods are mainly used in the field operation scene where the navigation line has been manually drawn, and it is difficult to solve the scheduling problems such as farm machinery back to the warehouse.
(2) When GNSS signals are occluded by objects such as trees, bridges, and buildings, there will be a situation of missing information. And GNSS also has certain errors due to the influence of signal time difference, ionosphere, and troposphere. This will have a great impact on the stability of the autonomous driving system.
(3) GNSS mainly provides location information for agricultural machinery. However, there are a large number of variable factors in the actual driving process of agricultural machinery, such as crop row may not be straight, crop lodging skew, and so on. GNSS technology cannot accurately perceive these ground specific environments, as shown in (Table 3).

**Table 3:** Technical difficulties in automatic navigation based on GNSS

Technical difficulties	Description
The complexity of rural road environment	The rural road environment is complex and changeable, and it is difficult to plan a safe navigation path without manually drawing navigation lines.
Signal blocking and error issues	1) Occluded by the surrounding environment, the problem of signal loss occurs. 2) Due to the influence of signal time difference, ionosphere, and troposphere, it is prone to errors.
Incapability to perceive variable factors	Variable factors such as non-linear crop rows and lodging deviation in driving cannot be accurately perceived.

The aforementioned problems can be reduced by studying the Beidou-ground-based augmentation system and inertial navigation^36^^,^^37^ elements to reduce the external electromagnetic interference and achieve 24 h of multi-angle work. Virtual Reference Station (VRS) technology can also be used to further improve the positioning accuracy of BDS. It reduces the real-time correlation error of occluded navigation signal in remote areas. The relative inertial navigation combined with differential equation recurrence formula is as follows.

(Equation 1) $${\dot{C}}_{m}^{s}=C_{m}^{s}\left[\omega_{ms}^{s}\times \right]=-C_{m}^{s}\left[\omega_{ms}^{m}\times \right]$$

(Equation 2) $$\left\{ \begin{array}{c} \omega_{ms}^{m}=C_{s}^{m}\omega_{is}^{s}-\omega_{im}^{m}\;\\ \left[\omega_{ms}^{m}\times \right]=C_{s}^{m}\left[\omega_{is}^{s}\times \right]C_{m}^{s}-\left[\omega_{im}^{m}\times \right] \end{array}\right.$$

Among them, $C_{m}^{s}$ represents the principal and sub-coordinate transformation matrix of inertial navigation system (INS) coordinate system. ${\dot{C}}_{m}^{s}$ is its differential. $\omega_{ms}^{s}$ represents the projection of the angular velocity of the sub-INS with respect to the master INS onto the sub-INS coordinate system. $\omega_{ms}^{m}$ represents the projection of the angular velocity of the sub-INS with respect to the master INS onto the master INS coordinate system.

The relative navigation differential equations are as follows.

(Equation 3) $${\dot{C}}_{m}^{s}=C_{m}^{s}\lfloor \omega_{im}^{m}\times \rfloor -{\lfloor \omega_{is}^{s}\times \rfloor C}_{m}^{s}$$

(Equation 4) $$\dot{V}=C_{s}^{m}f_{s}-f_{m}-2\omega_{im}^{m}\times V-{\dot{\omega }}_{im}^{m}\times R-\omega_{im}^{m}\times \left(\omega_{im}^{m}\times R\right)$$

(Equation 5) $$\dot{R}=U-\omega_{im}^{m}\times R$$

Here, ${\dot{C}}_{m}^{s}$ represents relative attitude differential equation. $\dot{V}$ is the relative velocity differential equation in the master node coordinate system. $f_{s}$ represents the accelerometer output of the child node. $f_{m}$ represents the accelerometer output of the master node. R represents the relative position. $\dot{R}$ is the relative position differential equation. U denotes the pseudo relative velocity.

The relative navigation error equation is as follows.

(Equation 6) $$\dot{\emptyset }=-\omega_{im}^{m}\times \emptyset -C_{S}^{m}\varepsilon$$

(Equation 7) $$\delta \dot{U}=C_{S}^{m}f_{s}\times \delta \psi -\omega_{im}^{m}\times \delta U+C_{S}^{m}\nabla$$

(Equation 8) $$\delta \dot{R}=\delta U-\omega_{im}\times \delta R$$

Here, $\dot{\emptyset }$ represents the differential equation of the relative attitude error. ε is noise. $\delta \dot{U}$ represents the pseudo relative velocity error. ∇ is the accelerometer noise. $\delta \dot{R}$ represents the relative position error.

### Automatic navigation technology of agricultural machinery based on machine vision

Machine vision^38^ is an automatic detection and analysis method based on imaging devices. It is usually applied in the industrial field. It provides technical support for applications such as automatic detection, process control, and automatic navigation of agricultural machinery. Machine vision has the advantages of rich information, high flexibility, wide range of vision, and low cost. Compared with other sensors, the operation of vision sensors is more convenient, which makes the automatic driving technology based on machine vision receive more and more researchers' attention. The automatic navigation technology of agricultural machinery based on machine vision is the process of understanding and perceiving the surrounding road environment, static and dynamic objects, and crop growth scenes during the driving process. It realizes the accurate positioning of agricultural machinery for its surrounding objects and scenes.

Environmental sensing technology^39^ is the basic link of agricultural machinery intelligence. It is also an important basis and primary link to realize a safer, more accurate, and more efficient automatic driving and autonomous navigation operation system of agricultural machinery. Agricultural machinery environmental sensing technology refers to the process of collecting, analyzing, and expressing the information in the environment of agricultural machinery during its driving and operation,^40^ so as to guide the safe and efficient operation and scheduling of intelligent agricultural machinery. In 1997, Gerrish^41^ tested autonomous navigation using a monocular vision system at speeds of 1.33 m/s and 3.58 m/s in different lighting environments with 95% confidence. And it can automatically navigate 125 m in the case of a continuous distance of 0.1 m from the crop, which greatly improves the accuracy of agricultural machinery field navigation. In 2005, Royer^42^ used a monocular vision system for automatic navigation in different lighting environments. The experimental results show that the average lateral error on the line is 0.018 m, and the maximum deviation on the curve is 0.161 m. In 2022, Ruangurai^43^ built a planter navigation system based on machine vision algorithms. He used Hough transform and principal-component analysis (PCA) to provide row spacing guidance and direction feedback for the planter. The experimental results show that the average error is less than 0.0398 m.

According to the existing research results, the perception techniques of rural road environment can be divided into feature detection algorithms and machine learning algorithms. The algorithm based on feature detection uses the color, texture, edge, shape, and other features in the image to find the possible driving area in the image.^44^ There are three common algorithms for environment perception based on feature detection. Method one, the color feature of the road is used to segment the image. Then, the wavelet texture feature fusion^45^ is used to fuse the oversegmented regions to achieve accurate road segmentation in complex environments. Method two, the Otsu method is introduced into the minimum error rate Bayesian decision to segment the image. Then the Hough transform^46^ is used for road and obstacle localization. Method three, the optical flow method is used to detect the moving obstacles in the video. Through the motion characteristics of the object and the ground, the possible obstacles in the video are found, and the update mechanism is used to reduce the false detection rate.^47^ However, the algorithm based on machine learning has better adaptability to environmental changes. By constructing and training a parameter model, the method can automatically learn the characteristics of the environment and finally complete the task of road and obstacle detection. There are four common environmental perception algorithms for machine learning. Method one: the image is segmented by superpixels, and the segmentation results are clustered. Then, a binary classifier based on k-nearest neighbor algorithm^48^ is used to classify the image into road and non-road regions. Method two: the multi-layer perceptron model^49^ is used to train the color and texture features extracted from RGB images, and the segmentation of rural roads is achieved. Method three: FCN^50^ is used to perform semantic segmentation of Mars ground images and satellite images. Aiming at the problem of variable outdoor light conditions, FCN is used to segment the drivable area in the multi-channel image after the fusion of near-infrared image^51^ and RGB image. Method four: proposed a rural road segmentation model SegNet^52^ based on Encoder-Decoder. The Dice similarity and Jaccard similarity coefficients can both reach more than 80%. This paper summarizes the aforementioned seven different machine vision algorithms and compares their advantages and disadvantages in automatic navigation, as shown in Table 4.

**Table 4:** Automatic navigation technology based on machine vision

Key methods	Basic principles	Characteristics
Hough transform^53^^,^^54^^,^^55^^,^^56^	Find the peak value in parameter space and determine the descriptive parameters of crop rows	It is suitable for the environment with high distinction between crops and soil and fewer weeds
Least squares method^57^^,^^58^^,^^59^	The regression operation of crop row feature points was carried out	The results are greatly affected by the feature extraction method, so the noise should not be excessive
Green pixel recognition method^60^^,^^61^^,^^62^^,^^63^^,^^64^	The ExG index of the image was calculated and the green crop rows were extracted	The calculation speed is fast, but it fails in the environment with more weeds
Full convolutional neural network^65^^,^^66^^,^^67^	The semantic segmentation of field scenes is carried out	It can achieve accurate crop row detection under different lighting conditions
Target centroid navigation line extraction algorithm^68^	The center line of the village is obtained by fitting the conic curve	The average deviation rate is 6.36%, and the performance is slightly worse on shaded and water-stained roads
Method of road extraction and navigation line generation^69^	HSV color space component weighted fusion, Otsu threshold segmentation road area and other areas	The maximum deviation of rural road is 0.216 m under the path condition of relief
UNet + ACBlock neural network^70^	Using ACBlock module to replace DACBlock module, the semantic segmentation of rural theory is carried out	The accuracy is 85.03%, and the image deduction speed is 2.7s/image

**Figure 3:**
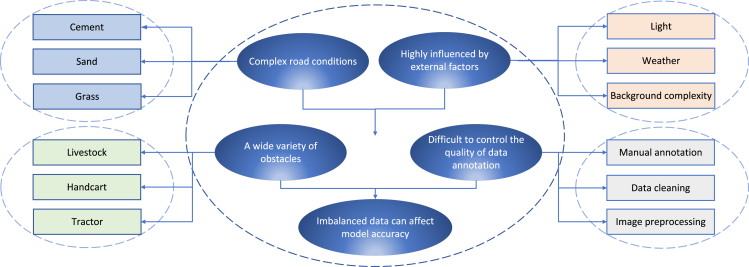
Technical difficulties in automatic navigation based on machine vision

Technical difficulties:

(1) Most of the paths of agricultural machinery automatic navigation are rural roads, and there are many kinds of drivable areas in the road, such as mud, grass, cement, sand, and gravel. And the road conditions are more complex than urban roads, including ups and downs, inclines, bumps, stretches and bends, and weeds cover, and the width of the road surface is variable. The method based on feature detection is easily affected by illumination, water on the road surface, background complexity, and other problems, resulting in a serious decline in performance.
(2) In the process of automatic driving of agricultural machinery, there are many kinds of dynamic and static obstacles in the drivable area. Static obstacles are relatively easy to distinguish, but dynamic obstacles have various poses, such as livestock, wheelbarrows, and tractors.^71^ For the environment perception technology based on machine learning, the requirements for visual sensors and model algorithms are relatively high. The model must learn deep feature information, otherwise its accuracy will not meet the standards of practical applications.
(3) Machine learning algorithms can effectively reduce the impact of environmental factors on model performance by supplementing corresponding data or data augmentation. However, most of the current models are based on supervised learning,^72^ and the image data need to be collected and manually labeled. The accuracy of the data directly affects the prediction results of the model.
(4) The image obtained by the vision sensor contains rich information, and the automatic driving system can perceive the shape of the road and obstacles through the image, which has a wide range of applications. However, visual sensors are easily affected by environmental factors such as illumination and weather. In the future, it is necessary to use a more robust deep learning model to replace the traditional manual feature extraction method.
(5) Machine-vision-based model algorithms require a large number of sample data to train. And the rural road environment is complex and changeable, and the construction of a data set requires manual classification and annotation of each category on each image, as well as manual screening and image preprocessing. Not only does this cost a lot of time, but the accuracy is also highly affected by the annotator of the dataset as shown in Figure 3.

**Figure 4:**
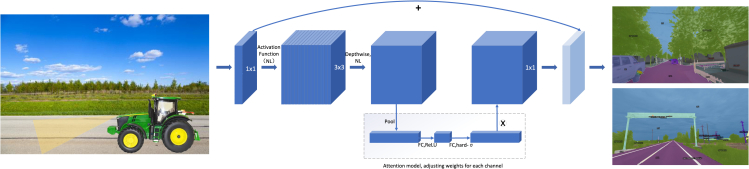
Rural road recognition based on machine vision

Most of the aforementioned methods use machine learning algorithms to assist the automatic navigation of agricultural machinery, which is not ideal for real-time inference of complex road conditions. Therefore, rural roads based on deep learning can be used for automatic navigation. Firstly, try to train an automatic annotation model to pre-label the target classification, and manually fine-tune the annotated images to reduce the workload. It can also calculate the position and attitude of the machine by adjusting the geometric relationship between the vision sensor and the object. Second, try to investigate network models such as EfficientPS^73^ or Transformer^74^ that perform end-to-end panoptic segmentation, and extend Deformable DETR^75^ to provide a unified mask prediction workflow for Things and Stuff, making the panoptic segmentation Pipeline^76^ more concise and efficient. Then, the fusion method of the feature layer in the neck network is improved. An encoder structure based on attention mechanism is used to better model the relationship between different feature layers and improve its adaptability to unstructured environments. It can also improve the prediction module of the model, design a panoptic segmentation prediction method based on mask classification, select the appropriate structure to decode the encoded features according to the prediction method, and construct the loss function and prediction matching principle required for model training. Through the classification of binary masks, postprocessing operations such as predicted label synthesis and non-maximal suppression are eliminated, the overall performance of the network is improved, and the end-to-end prediction of the network is further realized. As shown in Figure 4, taking the MobileNetV3 network as an example, images or videos can be taken through the visual sensors of agricultural machinery and input into the trained model for recognition. After a series of operations such as convolution and pooling, the recognized image is output. After training the model with a large number of samples, the model can correctly display the label of each category on the mask. This method can help agricultural machinery to identify which are the types of the current road surface, which roads can be walked, and which are the obstacles to be avoided, so as to realize real-time accurate automatic navigation.

### Automatic navigation technology of agricultural machinery based on laser radar

**Figure 5:**
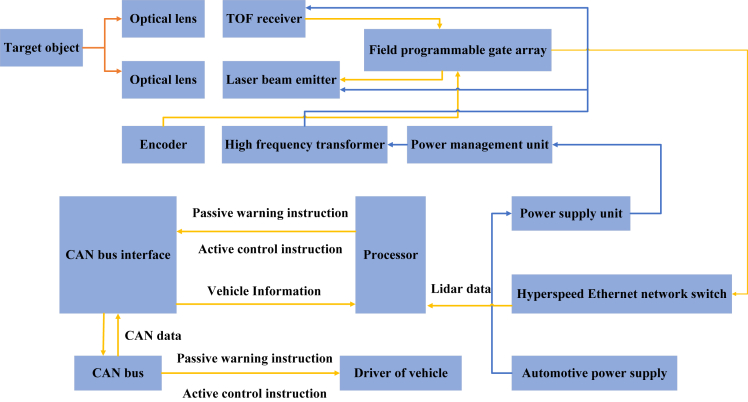
Laser Radar’s working principal

Lidar^77^ can be divided into two types: two-dimensional and three-dimensional. By sending the laser detection signal, receiving the signal reflected from the object to be detected, and calculating the time difference between the signals, the distance of the target can be obtained according to the speed of light. Lidar ranging technology has the advantages of long measurement distance, high accuracy, and strong anti-interference ability, which is mainly used in ranging and obstacle avoidance tasks. 3D lidar installed on agricultural machinery can achieve the reconstruction of 3D field point cloud image and obstacle detection.^78^ For example, the common TOF lidar is used in the task of obstacle avoidance in the automatic navigation of agricultural machinery. Its working principle is shown in Figure 5. In 360°, two optical lenses are used in turn to send a short but very high instantaneous power pulse of light through a laser emitter, and then the TOF receives the pulse and encodes it through a Field Programmable Gate Array (FPGA). Then the instructions are transmitted to the driver through the processor to obtain the scanning information. When the transmitted pulse speed is fast enough, real-time 360-degree lidar ranging and obstacle avoidance tasks can be carried out.

At present, more and more laser radar technologies are applied to the robot automatic navigation. For example, in 2018, Yang^79^ proposed a 3D laser scanning outdoor positioning method based on the "topology-geometry" map and the matching algorithm of 3D laser scanning points. Through field tests, the problem of automatic navigation obstacle avoidance is completed at the position of the minimum 1.8 m in the narrow channel, and the problems of poor GPS signal, low accuracy, and discontinuous output are solved. In 2020, Xiong^80^ used Hokuyo 2D lidar to realize the automatic navigation function of a single-rail multi-arm strawberry picking robot in the field. It mainly uses a topological navigation system^81^ to use a metric map and a chart together, which reduces the complexity of the automatic navigation environment. In 2022, Wang^82^ used 3D lidar to build an automatic navigation spraying system for orchards. Multi-source information fusion was carried out through millimeter wave radar to realize the perception of obstacles. The experimental results show that the emergency obstacle avoidance function can be achieved in the range of 0.5 m, and the navigation error is controlled within 0.15 m. Compared with Yang’s automatic navigation obstacle avoidance, the accuracy is improved, and the emergency obstacle avoidance function is added.

**Figure 6:**
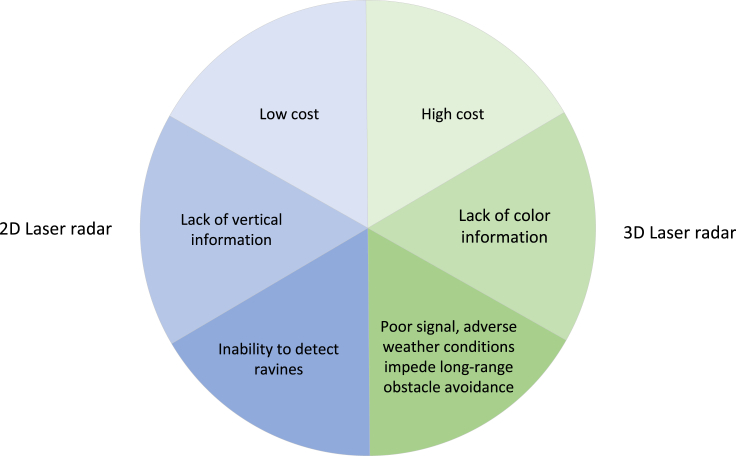
Technical difficulties in automatic navigation based on laser radar

Technical difficulties:

(1) The aforementioned applications are based on field operations, and the road conditions are relatively simple. However, the road conditions on rural roads are more complex, and there are too many dynamic and static obstacles to be identified to realize automatic navigation of agricultural machinery. For example, there will be gullies on both sides, and 2D lidar needs obstacles or fences on the side of the road to perform ranging.
(2) 3D radar can obtain more abundant road surface information, but high-precision 3D lidar costs too much, lacks color information, and its resolution is lower than RGB camera.
(3) In the rural road environment with bad weather or complex terrain, lidar is required to provide high-precision distance information and a powerful algorithm to generate the optimal path, which can avoid obstacles in time. Therefore, it is necessary for lidar to ensure real-time modification of the path of automatic navigation on the premise of having signals as shown in Figure 6.

Based on the aforementioned problems, try to change the location of the lidar installation, such as on the roof of the car to improve the visual range, detect more targets, and avoid collisions. It can also be mounted on the engine cover and embedded directly under the fog lights on the front side of the car. In this way, more information on both sides of the road can be obtained. In addition, multi-sensor fusion can be used according to different road scenes, such as camera-dominated and millimeter-wave radar to obtain more comprehensive road information. This approach can not only improve the reliability of environment perception but also reduce the cost at the same time.

In general, the aforementioned three navigation methods are the current mainstream agricultural machine automatic navigation methods. In order to ensure the accuracy and real-time performance of navigation, we can try to combine deep learning and computer vision technology. A variety of sensors including GNSS, inertial navigation system, and vision sensors are integrated to obtain road information at the same time. In addition, in the face of different terrain and environment, in order to ensure the high accuracy and real-time performance of agricultural machinery automatic navigation, it is necessary to consider the combination of more advanced algorithms to achieve obstacle avoidance function for agricultural machinery efficiently and quickly. Therefore, the next chapter introduces the path planning algorithm, discusses the differences and connections between different algorithms, and analyzes how to optimize the path planning algorithm in different scenarios. This method belongs to the last step of automatic navigation path generation, which can not only minimize the navigation time but also improve the accuracy of obstacle detection and ensure that agricultural machinery can generate navigation path normally, efficiently, and in real time in various environments.

## Research progress of path planning algorithms

Path planning algorithms are crucial steps in automatic navigation. In this paper, we propose the following four path planning methods for the rural road automatic driving environment of agricultural machinery. These methods can be applied to agricultural machinery according to different application scenarios and the advantages and disadvantages of each algorithm. For example, selecting a path planning algorithm that minimizes the cost of time and distance; the algorithm that adapted to different obstacle avoidance shapes, sizes, and positions; and algorithms with constraints on the speed and energy consumption of agricultural machinery. In a dynamic environment, choosing different real-time planning algorithms to avoid constraints such as moving targets can make the automatic navigation of agricultural machinery more efficient. Therefore, this paper compares the path planning methods based on graph search, sampling, optimization, and learning, which contains the basic ideas of 22 path planning algorithms, and gives a detailed explanation of their basic principles.

### Path planning method based on graph search

**Figure 7:**
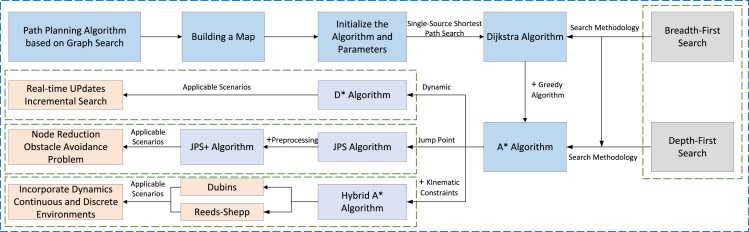
Relation diagram of path planning algorithms based on graph search

The choice of path planning method is the most important for the accuracy of automatic navigation of agricultural machinery. Among them, the path planning methods based on graph search include Dijkstra algorithm, A∗ algorithm, Hybrid A∗ algorithm, Jump Point Search (JPS) algorithm, JPS+ algorithm, and D∗ algorithm. These methods can be used to convert the road network into the form of a graph and find the shortest path from the start point to the end point. The basic relationship and applicable scenarios of the six algorithms are shown in Figure 7.

Among them, Dijkstra algorithm uses the idea of greedy algorithm to find the shortest path for a node at each step, which is Breadth-First Search (BFS) single-source shortest path. In 2020, CHE^83^ used the Dijkstra algorithm combined with polygon fitting and hierarchical polyhedron fitting methods to complete the two-dimensional and three-dimensional path planning of the underwater vehicle with fixed depth and realized the ROV operation of the underwater vehicle. The A∗ algorithm has two search methods, BFS and Depth-First Search (DFS). DFS diverges around the starting point as the center of the circle, searches all the points around it in the form of a circle, and if no point is found, it continues to expand the radius until a point enters the target area. BFS is to make the starting point as far away from the search area as possible, as close as possible to the searched point, and then slowly spread. Compared with Dijkstra algorithm, A∗ algorithm reduces the number of sampling grids and increases the path search speed. In 2020, Zhong^84^ combined A∗ algorithm and adaptive window algorithm to simplify the calculation of risk cost function and distance cost, reduce the number of grid nodes, and realize smooth path tracking. In 2022, Li^85^ used lidar to build a map and combined the improved A∗ algorithm with the DWA algorithm to realize the global path planning and real-time local path planning of plant protection operations in dwarf and dense jujube orchard. The U-shaped path exhibits an average error of 0.0269 m, whereas the linear path demonstrates a displacement error of 0.0247m. Additionally, the L-shaped path showcases a navigation error of 0.0268 m. These results indicate its capability to effectively address the path planning challenges encountered by agricultural robots navigating and positioning themselves on rural roads. The distinction between the Hybrid A∗ algorithm and the A∗ algorithm lies in its consideration of the kinematic constraints of the vehicle during path planning, ensuring adherence to maximum curvature limitations, and predominantly employing Dubins curves and Reeds-Shepp (RS) curves for trajectory generation. In 2010, Dolgov^86^ first used autonomous vehicles for path planning on unstructured roads and combined the 3D kinematic state space of the vehicle with the A∗ algorithm to obtain the running trajectory. The quality of the solution is enhanced through numerical nonlinear optimization, resulting in a planning period for a complete vehicle running trajectory ranging from approximately 50 to 300 ms. JPS algorithm is also based on the improvement of A∗ algorithm, which is more suitable for obstacle avoidance in path planning. In the path planning, by considering the minimum cost of passing through the obstacles, these minimum cost points are used as the starting point of the next search path. Compared with the previous algorithms, the search efficiency will be improved more, and the number of points in the open list will be reduced. That is, some hop points are fixed during the search process to reduce the amount of path exploration by jumping unnecessary grid points. In 2021, Hu^87^ proposed a combination of JPS+BB (Bounding Box) on the basis of JPS algorithm to perform single-source canonical Dijkstra operation on each traversable node in the grid. JPS+ algorithm is based on JPS algorithm, adding preprocessing operations to avoid the problem of excessive depth caused by multi-layer recursion. In 2020, Jiang^88^ proposed a JPS+ algorithm using bidirectional alternating orthogonal search mechanism. The algorithm not only accelerates the search speed but also ensures the safety of the mobile robot in the process of motion. In 2005, Likhachev^89^ proposed D∗ algorithm for the first time. D∗ algorithm is actually a dynamic A∗ algorithm, which belongs to incremental path planning algorithm. Compared with the A∗ algorithm, the D∗ algorithm is a heuristic search path algorithm, which is compatible with static environments and dynamically changing scenarios. And instead of searching from the starting point to the target point, it uses the idea of backpropagation to search from the target point to the starting point to find the shortest path to the starting point. This method was first used in the Mars rover for robotic pathfinding, and it is suitable for searching paths in unknown environments or with dynamic environmental changes.

**Figure 8:**
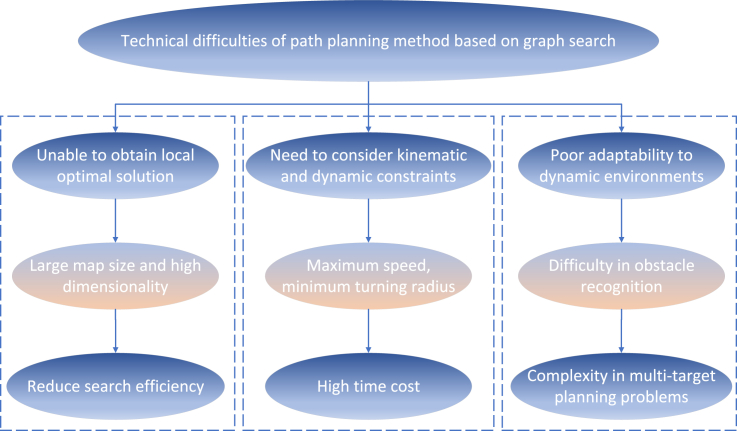
Technical difficulties of path planning method based on graph search

Technical difficulties:

(1) The path planning algorithm based on graph search can generally find the global optimal solutions, but when the map is too large, the dimensions of the paths to be planned become too high, which will reduce the search efficiency.
(2) For the agricultural machinery that needs to plan the real-time path to avoid collision, it is relatively difficult to calculate an optimal path with the maximum speed and minimum turning radius considering the kinematic and dynamic constraints, which will increase the time cost of algorithm search and planning.
(3) The method based on graph search is generally more suitable for static environments and have limited adaptability in dynamic environments. With the change of time, the difficulty of recognizing dynamic obstacles increases significantly, especially when multiple targets appear in the field of view of agricultural machinery, making path planning extremely complex as shown in Figure 8.

Based on the aforementioned problems, we can first try to use the Lazy strategy, which does not detect obstacles when sampling points and generating paths and directly performs path planning on the generated graph to improve efficiency. Or a problem is divided into multiple sub-problems, combining global path planning and local path planning to solve different challenges. First, the global path planning is used to formulate the approximate path, and then the local path planning is used to avoid obstacles in each segment of the path of the agricultural machinery. Another approach is to combine with sensors to solve the problem of dynamic environment changing. By periodically detecting changes in the environment using the sensor data, the agricultural machinery can continuously re-plan the path effectively.

### Path planning method based on sampling

Sampling-based path planning includes Probabilistic Road Map (PRM) algorithm, Rapid-exploration Random Tree (RRT) algorithm, Kinodynamic RRT∗ algorithm, and Informed RRT algorithm. Through these sampling techniques, a set of candidate routes can be generated, and then the optimal route can be selected by combining the distance length, road capacity, and vehicle speed through the evaluation function. The connections and differences of these four algorithms are shown in Table 5. The advantage of sampling-based path planning methods over graph search methods is that the entire configuration space and boundaries do not need to be explicitly constructed.

**Table 5:** The path planning algorithm based on sampling

Algorithm	Sampling method	Characteristics	Applicable scenarios
PRM	Random point sampling	Through random pick points, generate roadmap, comparing the cost and threshold, to find the optimal path.	Multi-objective, multi-constraint problem
RRT	Random tree sampling	Through random extension and connection nodes generated random tree, generate a path from the starting point to find target point.	Large-scale, high-dimensional, single-objective problem
Kinodynamic RRT∗	Sampling with curves instead of straight lines	By changing the straight line connection between two random sampling points into a curve connection, the smoothness of the path is increased, and the optimal path is generated by combining the dynamic characteristics.	Large-scale, high-dimensional, complex environmental problems based on dynamic constraints
Informed RRT	Ellipse sampling	Constructed a three-dimensional ellipsoid coordinate system, and a heuristic function is used to guide the growth direction of the random tree to achieve rapid expansion	Single-source rapid obstacle avoidance problem

In 2010, Baumann^90^ proposed the PRM algorithm, which collected points in the form of random sampling, generated point-to-point straight lines by comparing cost and threshold, built a roadmap, and finally sought the optimal path with the lowest cost. In 2019, Mahmud^91^ designed an algorithm to solve the problem of automatic navigation of a pesticide spraying robot in a greenhouse based on the PRM path planning algorithm, which was used to generate a path between plants and determine an optimal path with minimum navigation cost for the robot. Different from PRM algorithm, RRT algorithm takes the starting position of the search as the root node and increases the leaf nodes by random sampling. A random tree is then generated, and the path from the starting point to the goal position can be drawn once the goal region contains leaf nodes. In 2021, Li^92^ combined RRT-Connect and RRV algorithm to propose an adaptive RRT-Connect (ARRT-Connect) algorithm, which is used to solve the problem that the robot is difficult to solve when it encounters a narrow channel and makes it have strong environmental adaptability. It can complete the optimal path planning in a short time. Kinodynamic RRT∗^93^ algorithm is an improvement based on RRT algorithm. RRT is mainly a direct connection between two points, which is not smooth. Kinodynamic RRT∗ mainly connects x_near and x_new by replacing the straight line in the algorithm with a curve, so that the generated path is more in line with the dynamic constraints and meets the dynamic requirements of the robot. In 2020, Hu^94^ also proposed a Kinodynamic-based RRT algorithm, which uses a straight line to connect a pair of sampled waypoints, so that the robot can reach the destination in a short time without collision. The Informed RRT^95^ algorithm is a further innovation, which accelerates the optimization speed by randomly adding ellipse sampling constraints. In two-dimensional space, the ellipse range is constructed by taking all lines of the starting point as the major axis of the ellipse. In three-dimensional space, an ellipsoid is directly constructed, and the path is constantly corrected by the bias of coordinate system transformation. But this method is more suitable for solving the path planning problem of single-source fast obstacle avoidance.

**Figure 9:**
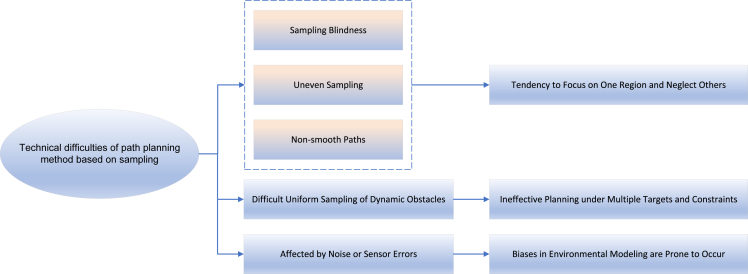
Technical difficulties of path planning method based on sampling

Technical difficulties:

(1) Sampling-based path planning is prone to problems such as sampling blindness, uneven sampling, or unsmooth path. These sampling biases can make the algorithm focus on a certain region and ignore other regions.
(2) The planning effect of sampling-based path planning algorithm under multi-objective and multi-constraint conditions is not very good. Especially in the dynamic environment, when the obstacles keep changing positions, it becomes a challenging task to sample points uniformly and dynamically.
(3) This method needs to model the environment accurately to recognize the positions of obstacles and targets. Large modeling biases can occur if there is significant noise in the surrounding environment or sensor errors as shown in Figure 9.

One possible approach is to combine this method with a greedy algorithm. When obtaining a new node, check if it can directly reach the target point. Alternatively, change the search scope of potential optimal parent nodes from the node tree to the constructed path, reducing the number of nodes to be searched and improving navigation efficiency. It is also possible to use hierarchical sampling to divide the state space into multiple subregions for fast search at the coarse level and more refined search at the detail level. Alternatively, Gaussian Mixture Model (GMM)^96^ is combined to model the distribution of the state space, which captures the structure and change in the state space more comprehensively. Another method is to continuously repeat sampling in some important areas according to the progress of path planning and improve the sampling density in this area, which can reduce the deviation problem of sampling. The aforementioned methods can not only be used alone but also try to fuse multiple methods to be used together according to actual application scenarios to meet various requirements of path planning.

### Path planning method based on optimization

**Figure 10:**
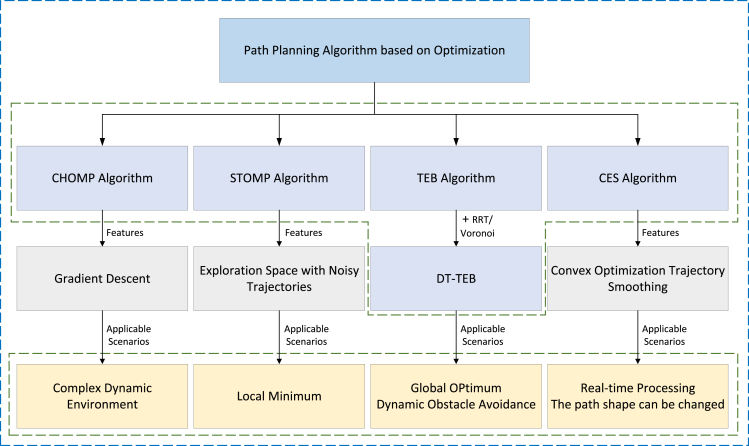
Diagram of the path planning algorithm based on optimization

Optimization-based path planning includes Covariant Hamilton Optimization Motion Planning (CHOMP) algorithm and Stochastic Trajectory Optimization Motion Planning (STOMP) algorithm, Timed Elastic Band algorithm (TEB), TEB in distinctive topologies algorithm (DT-TEB), and Convex Elastic Smoothing (CES) algorithm. These methods are the process of transforming the path planning problem into an optimization problem and then optimizing the solution. The principle is to transform the multi-objective, multi-variable, and multi-constraint coupled programming model into a weighted penalty function, and the differences and connections of these five algorithms are shown in Figure 10.

Among them, CHOMP algorithm is a gradient-based trajectory optimization method. It uses the method of covariant gradient and function gradient in the optimization phase to create an initial path from the starting position to the ending position and uses the gradient descent method to optimize the trajectory for the cost function. In case the initial trajectory is not feasible, the CHOMP algorithm can change the path trajectory according to the surrounding environment. At the same time, dynamic quantities such as joint velocity and acceleration are optimized, so that it can quickly converge to a smooth collision-free trajectory and improve the execution speed of the robot. In 2013, Zucker^97^ proposed this method and applied it to the seven-degree-of-freedom manipulators and a rough-terrain quadruped robot. It uses the Hamiltonian Monte Carlo method instead to alleviate the problem of convergence to a high-cost local minimum. Experiments show that the method can converge to a low-cost trajectory even when initialized as an infeasible trajectory and perform strictly along the trajectory. However, this method is easy to fall into the local minimum problem in gradient descent, whereas the STOMP algorithm proposed by Kalakrishnan^98^ can explore the space around the initial trajectory by using a trajectory with noise. And this method does not need the gradient information of the objective function, so it has strong randomness. The proposed method can not only overcome the local minimum problem generated by gradient descent methods but also reduce the cost of trajectory exploration. The TEB^99^ algorithm belongs to the local path planning algorithm, which is able to correct the initial trajectory generated by the global path planner. The principle is to describe both dynamic and static constraints and the robot state using a sparse graph with strong expansion properties. Then it is transformed into a weighted single-objective optimization problem, and finally, the least square method is used to find the minimum cost path conforming to the constraints. This method is suitable for various types of wheeled robots such as various differential, omnidirectional, and Ackermann models and has a strong perspectiveness, and the effect of obstacle avoidance and path replanning for dynamic obstacles is very good. However, the time complexity of this method is relatively large, the fluctuation of velocity and angular velocity is relatively large, and it is not global optimal. Therefore, the same author made a further improvement on TEB algorithm in 2017, which is called DT-TEB.^100^ By fusing RRT algorithm with Voronoi diagram and TEB algorithm, respectively, this method maintains and optimizes candidate trajectories of different topologies and generates the global optimal trajectory in real time using the local topology space. By using dual variable Karush-Kuhn-Tucker (KKT) multipliers, the nonlinear path planning problem is transformed into an approximate nonlinear least squares optimization problem. Therefore, it not only prolongs the length of the trajectory generated by the algorithm but also improves the performance of the model obstacle avoidance. Marin-Plaza^101^ analyzed the performance of TEB algorithm based on Ackermann model and tested it on the Intelligent Campus Automobile (iCab) platform of Carlos III University. It is proved that the method and the various modules related to navigation can coexist and reach the target point without collision. CES^102^ algorithm is a convex optimization trajectory smoothing method, which generates a collision-free space tube through a series of bubbles and then constructs a convex optimization space by planning the velocity and path shape. Finally, the optimization of the shape and speed of the path is completed in tube by iteration until the trajectory converges and no longer changes. This method is suitable for real-time processing systems, has good adaptability to the input reference trajectory, and the calculation time is usually within 1 s.

**Figure 11:**
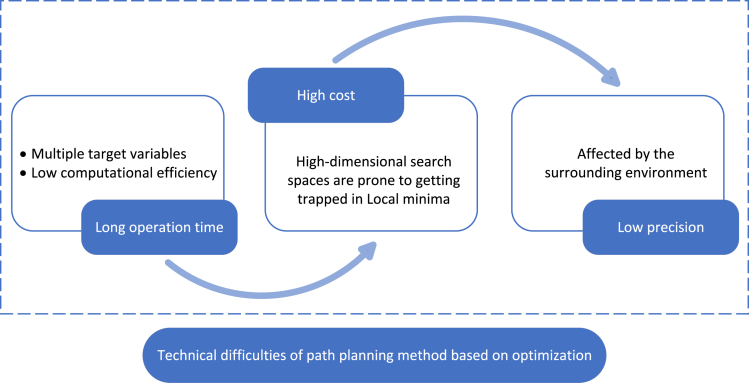
Technical difficulties of path planning method based on optimization

Technical difficulties:

(1) The advantages of the path planning method based on optimization lie in their ability to handle a variety of soft and hard constraints and ensuring the continuity of the curve. However, compared with the previous two methods, these algorithms require longer computation time when optimizing multiple object variables.
(2) When dealing with high-dimensional search spaces in this approach, algorithms become more complex, making it easier to converge to local minima rather than global minima, and the optimization cost is generally higher.
(3) For navigation scenarios with high precision requirements, the effectiveness of this method in path planning is limited. It is susceptible to environmental influences, and the computational efficiency of the algorithm will be reduced significantly as shown in Figure 11.

One approach to address these challenges is to consider using multi-objective optimization algorithms such as Non-dominated Sorting Genetic Algorithm II (NSGA-II)^103^ or multi-objective evolutionary algorithm based on decomposition (MOEA/D).^104^ Under the premise of considering the path length, computation time, and navigation safety of agricultural machinery automatic navigation, genetic algorithm and simulated annealing algorithm can be combined to gradually approach the optimal solution by constantly searching and accepting bad solutions. Alternatively, the principal-component analysis (PCA)^105^ method is used to map the high-dimensional space to the low-dimensional space to reduce the dimensional complexity of path planning, and the Bayesian optimization algorithm is combined to deal with the noise that may be caused by the surrounding environment and reduce the error of path planning. Another approach is to utilize multi-core processors for distributed computing to accelerate the speed of path planning, and use model pruning strategy to reduce the search space required for path planning, so as to improve the computational efficiency of generating the optimal path.

### Path planning method based on learning

Learning-based path planning includes methods such as deep learning and reinforcement learning. All of them learn experience from existing data through machine learning methods, find out the corresponding functional relationship, and let the machine have the ability to learn, so as to formulate path planning strategies.

The difference is that deep learning is actually supervised learning, high-dimensional data are mainly transformed into low-dimensional data features by deep neural network (DNN),^106^ convolutional neural network (CNN),^107^ and recurrent neural network (RNN).^108^^,^^109^ The path planning strategies learned in the past are summarized, and the previous similar strategies are selected according to the current application scenario to achieve the purpose of learning. In particular, convolutional neural networks are more and more widely used in large-scale image recognition, speech recognition, and natural language processing. With the continuous enhancement of image processing units, there are various methods to solve the path planning problem using its deep network. In 2021, Zhang^110^ realized the shortest path planning of corn weeding robot in the field based on the twice traversal algorithm combined with Faster R-CNN network. And the improved OTSU algorithm improves the speed of path search and reduces the model calculation processing time. The algorithm achieves 90% path search success rate on the test data, which not only helps farmers improve the efficiency of weeding but also effectively protects the crops in the field. In 2022, Liu^111^ proposed a Res-Planner method for the real-time path planning problem of UAV. The residual convolutional neural network is used to train the information such as path state and behavior generated by the traditional static path planning algorithm, so as to achieve the purpose of real-time path generation. Experimental results show that the method can generate the global optimal path under the limited environmental information.

Reinforcement learning is to randomly and dynamically exchange data between agents and the environment through interactive learning. It is neither supervised learning nor unsupervised learning. It can learn with a relatively small amount of data, strengthen the training of the strategy with good results, and adjust the path planning strategy at each step through a self-learning process. That is, the best path planning strategy is achieved by constantly modifying the scheme to adapt to the environment. Generally, it includes four elements: policy function, value function, reward function, and environment model. In 2015, Zhang^112^ proposed a Geometric Reinforcement Learning (GRL) algorithm, which used the specific reward matrix for multi-UAV path planning. The optimal path is calculated in terms of path length and risk measure and adaptively update the current path according to the geometric distance and risk information shared by other UAVs. In 2022, Yang^113^ proposed a Residual-like Soft Actor Critic (R-SAC) algorithm based on reinforcement learning to solve the problem of robot path planning for agricultural scenarios. It can realize safe obstacle avoidance and intelligent path planning of agricultural robot in dynamic and static obstacle environment.

In addition to using deep learning or reinforcement learning methods alone, some scholars have designed the Deep Reinforcement Learning (DRL) methods by combining the two methods. This method not only has the perception ability of deep learning but also has the decision-making ability of reinforcement learning, which is a method closer to human learning thinking. In 2020, Gao^114^ combined the Twin Delayed Deep Deterministic policy gradients (TD3) algorithm in DRL method with the PRM algorithm to design a new PRM+TD3 path planner and used the incremental training method to solve the problem of path planning for mobile robots. In 2021, He^115^ also used DRL to train a path planner and designed an interpretable path planning algorithm based on DNN. By combining CAM and SHAP saliency map generation method, the problem of autonomous flight of quadrotor in unknown environment is solved.

**Figure 12:**
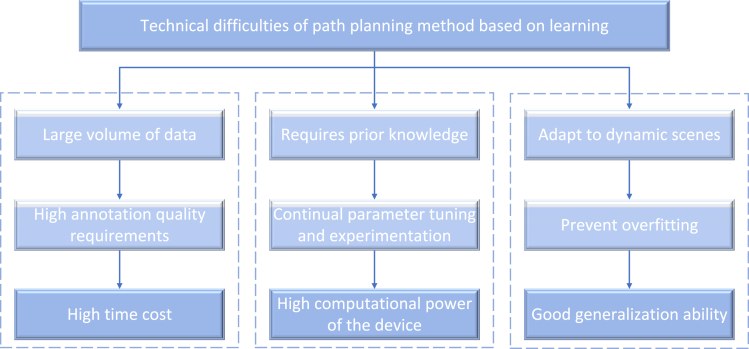
Technical difficulties of path planning method based on learning

Technical difficulties:

(1) The path planning method based on learning can generally achieve high-precision path planning, but it needs a huge amount of data support and requires high quality of data annotation, which will cost more time. If the quality of the annotation is poor, the performance of the learning algorithm will be affected.
(2) The calculation of learning-based path planning algorithm is generally large, and the parameters need to be constantly adjusted according to the model selected for learning, so that it can formulate the optimal path according to different scenarios. And for different thresholds, if there is no prior knowledge, it is likely to be reverse tuned, which may require multiple training iterations and trial-and-error, so it requires high computing power of the required equipment.
(3) The higher the accuracy of automatic navigation, the higher the safety of driving agricultural machinery. Therefore, the deep learning model is required to have strong generalization ability to ensure that the model can plan the path better and faster in new environments and dynamic scenes. In addition, we should prevent the problem of overfitting the model and prevent too much noise from causing too much deviation of the model generation path as shown in Figure 12.

To address these issues, we can try to use data augmentation methods, such as rotation, translation, flipping, scaling, shearing, and adding noise, to expand the amount of data for the model to learn and train and improve the generalization ability, or using a deeper neural network, given some specific parameters in the target scene, let the model learn some more obvious features to improve the environmental adaptability of the model. The reinforcement learning algorithm can also be combined with the greedy algorithm, the original features can be encoded by the reinforcement learning algorithm, the probability of not-selected nodes can be predicted through the attention mechanism, and then the greedy algorithm can be used to decode, so as to improve the accuracy of path planning.

## Discussion

In recent years, researchers from various countries have conducted extensive research in areas such as GNSS-based navigation, machine vision-based navigation, Lidar-based navigation, and agricultural machinery path planning, achieving certain results that have laid a solid foundation for this study. However, research and development in the field of automatic navigation for agricultural machinery in rural road scenarios has progressed relatively slowly, and there are several deficiencies and challenges that urgently need to be addressed.

(1) For GNSS-based automatic navigation of agricultural machinery, the establishment of GNSS base stations in urban areas is already well developed. However, in remote rural areas, especially in field locations, GNSS signals may be relatively weak, which could lead to signal loss and interruptions. Therefore, there is an urgent need to research and develop GNSS receiver technology with stronger signal reception capabilities to ensure real-time automatic navigation of agricultural machinery in various geographic environments. Additionally, the integration of other sensor technologies is necessary to enhance positioning accuracy.
(2) At present, the automatic navigation of agricultural machinery based on machine vision is mostly based on image processing technology and traditional machine learning algorithms to construct the drivable area calibration model and obstacle detection model, respectively. Although it is efficient and simple, it needs to combine a large number of people’s prior knowledge of environmental characteristics, and the results are low precision, low robustness, strong subjectivity and pertinency, and difficulty to reproduce. Moreover, there are many environmental variables in the actual driving process of agricultural machinery, so a lot of model modification work needs to be done, and sometimes other types of sensors besides images need to be combined in order to achieve the generalization of the model for unstructured rural roads. At present, some useful exploration based on deep learning has been carried out for the research of agricultural machine automatic navigation based on machine vision, but the existing research is still in the initial stage of exploration.
(3) Lidar automatic navigation technology is currently mainly focused on the mapping of farmland and farm terrain when agricultural machinery works in the field, which can help agricultural machinery avoid obstacles and plan paths. However, there are still few related studies on agricultural machinery driving on rural roads, and there are many restrictions on the drivable area detected by radar. The detection effect of static obstacles such as trees and buildings is good, and the real-time detection of dynamic obstacles is relatively low. Moreover, due to the high cost of lidar, it is generally applied to small farms, and it is also necessary to focus on the development of lower cost lidar technology. At the same time, it is necessary to strengthen the research of anti-jamming radar technology in different weather environments, so that the agricultural machinery can navigate normally and automatically under the weather conditions of rain and snow, fog, and strong light and reduce the time cost.
(4) Lack of deep learning image models designed for farmland and outdoor scenes. At present, most of the general models in the field of urban street scene recognition and perception are used. The difference between rural road scene and urban street scene is large, and the general deep learning model has strong non-structure, and the direct application effect in these unstructured scenes is poor. Existing research mainly uses semantic segmentation models to complete field environment perception tasks in hilly and mountainous areas. However, the model can only distinguish image content of different semantic categories but cannot distinguish different individuals of the same category and cannot recognize rural road scenes. In the rural road scenario, it is crucial to distinguish different instances of the same class of objects. The characteristics of hilly and mountainous environment are quite different from those of rural road environment. In addition, previous studies only focused on man-made paved roads and did not consider extreme conditions such as sand and dirt roads, so that the model could not be generalized to the rural road environment involved in this study.
(5) A large number of researchers have studied the two tasks of drivable area detection and obstacle detection separately and lack a unified model architecture to solve the two problems simultaneously. This is bound to complicate the image preprocessing work and the prediction label fusion work when the two tasks are performed at the same time. At the same time, the huge amount of computation brought by multiple models will bring huge obstacles to the real-time scene perception task of agricultural machinery.
(6) At present, there is existing research on automatic navigation path planning for urban roads and fieldwork robots. However, research on path planning for rural roads is relatively limited. The complexity of rural roads increases the difficulty of path planning algorithm, especially in the problem of obstacle avoidance. It is necessary to avoid dynamic and static obstacles and realize real-time automatic navigation while selecting the optimal path. In the future, multi-task learning can be attempted to integrate different terrain, road information detection in drivable areas and obstacle detection into a unified model to improve the speed of model calculation. And it is necessary to develop a path planning algorithm that is not affected by weather, geographic conditions, obstacle movements, and variations in plant growth. This will ensure real-time automatic navigation of agricultural machinery while reducing energy consumption.
